# Genetic Diversity and Population Structure of *Hemiculter leucisculus* (Basilesky, 1855) in Xinjiang Tarim River

**DOI:** 10.3390/genes13101790

**Published:** 2022-10-04

**Authors:** Siyuan Sun, Zhenyi Hu, Zhengyi Lu, Lu Liu, Xuan Liu, Qiong Zhou, Bin Huo, Dapeng Li, Rong Tang

**Affiliations:** College of Fisheries, Hubei Provincial Engineering Laboratory for Pond Aquaculture, Huazhong Agricultural University, Wuhan 430070, China

**Keywords:** Tarim River, SSR marker, Species invasioFigure, population genetic structure

## Abstract

*Hemiculter leucisculus* is an invasive fish and widely distributed in the Xinjiang Tarim River. In this study, RAD-seq was used to explore the genetic diversity and population subgroup structure of *H. leucisculus* in the Tarim River and develop relevant Simple Sequence Repeat (SSR) markers. The study collected 40 samples distributed at four different sites of the Tarim River. A total of 7,291,260 single nucleotide polymorphisms (SNPs) were obtained. The genetic diversity results showed that the population genetic diversity level of *H. leucisculus* was low. The population pairwise *F*_ST_ values ranged from 0.231 to 0.258, indicating that there was moderate genetic differentiation among these populations. AMOVA showed that the genetic variation within populations accounted for 92.31% of the total variation. The principal component analysis (PCA) and neighbor joining (NJ) tree revealed that the four populations could be separated into two clusters (upper-middle and downstream populations) and the individuals from Taitema Lake (TTMH) showed differences and had a bigger geographic distance than the others. There is the probability that the *H. leucisculus* from Bosten Lake entered Taitema Lake to breed and then expanded into the Tarim River due to the water diversion projects in location. In addition, 147,705 SSRs loci were detected and 22,651 SSR primer pairs were developed. This study will contribute to providing valuable molecular data for the management of wild populations, marker-assisted selection and resource exploitation of *H. leucisculus*.

## 1. Introduction

*Hemiculter leucisculus* (Basilewsky, 1855) is a small cyprinid fish. It has a wide distribution in East Asia, living in the upper middle layer of water and likes to live in clusters. It is an invasive fish in the Tarim River of Xinjiang. In the past, the economic value of *H. leucisculus* was considered low, so its potential value has not been exploited. However, in some areas where stable populations have been established, it has become an important economic fish [1]. Xie Zongyong et al. studied *H. leucisculus* of Fenhe Reservoir and the growth characteristics of *H. leucisculus* [2]. Li Baolin et al. conducted biological research on *H. leucisculus* from Dalai Lake [3]. Currently, the research on *H. leucisculus* mainly focuses on classification, nutrition and biology. At the same time, there is less research on the genetic background of *H. leucisculus*, which makes it difficult to gain strong theoretical support for the population dynamics of *H. leucisculus*. Therefore, it is important to study the genetic diversity of *H. leucisculus*.

RAD-seq (Restriction site-associated DNA sequencing) is a series of sequencing technologies based on restriction endonucleases characterized by simple operation, low application cost and wide coverage. It provides an accurate and efficient technique for studying population genetic structure and diversity at the genome level and has been widely used in these fields. Microsatellites, also known as simple sequence repeats (SSR), are abundant and dispersed broadly in the coding region and non-coding region of eukaryote genome and are composed of short (2–5 bp) variable tandemly repeated arrays. SSR markers are very practical genetic markers widely used in many fields, such as paternity testing, molecular marker-assisted breeding, genetic map construction, quantitative trait loci location and population genetics [4,5].

In this study, we used the RAD-seq method to unravel the population structure of *H. leucisculus* in the Tarim River. The objectives of our study were to use SNP genotyping to investigate and evaluate the genetic diversity, genetic differentiation and population structure of *H. leucisculus* and develop SSR markers. These data are the basis for the formulation of effective management strategies for this invasive fish, providing essential information both for its control and its potential exploitation as a fishery resource.

## 2. Materials and Methods

### 2.1. Sampling and DNA Extraction

**Figure 1:**
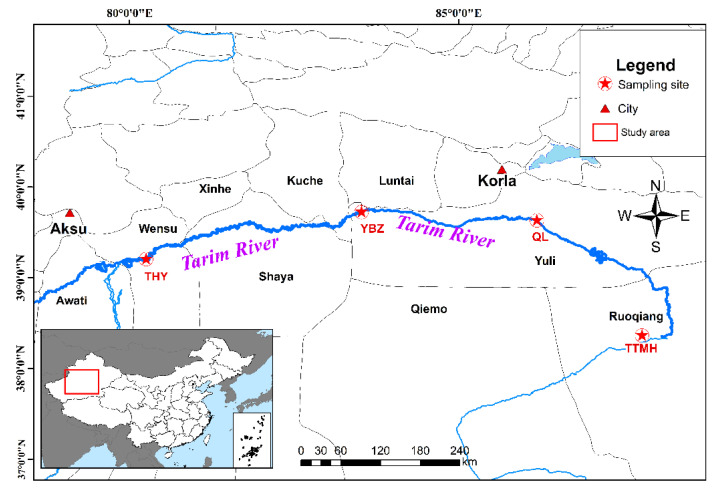
Sample sites of *H. leucisculus* in the Tarim River basin located in Xinjiang.

Along the 1321 km long main course of the Tarim River, fish were sampled in four sectors corresponding to the upper, middle and lower reaches of the river. Fish samples were taken from four locations in the Tarim River basin from May to October 2019. In total, 40 specimens were collected from four sample sites in the Tarim River basin (Figure 1). The fin samples were obtained and stored in 95% ethyl alcohol in −20 °C refrigerators for subsequent experiments. The genomic DNA of 40 individuals was extracted using the CTAB (Cetyltrimethylammonium Bromide) method. DNA quality and concentration were measured by 1.0% agarose gel electrophoresis and NanoDrop 2000 spectrophotometers (Thermo Scientific, Wilmington, DE, USA).

### 2.2. RAD Library Construction and Sequencing

The restriction site-associated DNA sequencing library was constructed using the recommended method described by Willing E M et al. (2011) [6]. About 1 μg of genomic DNA per sample was digested with *EcoR*I and incubated at 37 °C with T4 DNA ligase and *EcoR*I adapter. Using NEBNext dsDNA fragmentase, the reaction mixtures were segmented into 50–1000 bp. The samples were purified with AMPure XP beads (Beckman Coulter, Brea, CA, USA). The fragmented DNA was used to conduct end repairing, dA-tailing and adapter ligation. Subsequently, the PCR productions were purified and pooled to obtain the lengths of 300–400 bp digested fragment sequences for library construction. PCR amplification was performed on the size-selected fragments (12 cycles of PCR; 50 μg reaction system) using NEBNext high-fidelity 2X PCR master mix. Finally, the PCR products were purified using a High Sensitivity DNA assay Kit (Agilent Technologies, Lithuania) and sequenced on the Hi-Seq 2500 platform (Illumina Inc., San Diego, CA, USA). 

The raw data generated from the Illumina sequencer were filtered with the fastp program. Low-quality data filtering included removing reads with adapter, reads containing more than 10% of N and reads containing more than 50% bases with a quality value of less than 20 to obtain clean reads for subsequent bioinformatic analysis. To ensure the quality and quantity of the data, the minor allele frequency (MAF) was below 0.01. With the population RAD-tags set as a reference, the Burrows–Wheeler Aligner (BWA) was used to compare reads of each sample in the population, and GATK, a mutation detection software, was used to detect SNP in the population.

### 2.3. Genetic Diversity and Population Structure

SNPs obtained by sequencing were used to evaluate population genetic statistics, including average nucleotide diversity (Pi), observed heterozygosity per locus (*H_o_*), expected heterozygosity (*H_e_*), and Wright’s inbreeding coefficient (*F*_IS_). Pairwise F-statistics (*F*_ST_) among the four populations were performed using the Arlequin v3.5 [7]. In addition, to detect within and among populations genetic variance components, molecular variance (AMOVA) was analyzed using the StAMPP v1.6.3 [8].

A neighbor-joining (NJ) tree was constructed to cluster the populations using TreeBest software. Based on SNP differences between individuals, principal components analysis (PCA) can cluster individuals into different subgroups according to different traits. PCA analysis was conducted by software GCTA v1.93.2 [9], and the scatter plotting was performed with the first and second components using the Ggplot2 package [10] in R. The Admixture software [11] was used to constructed population structure in order to analyze the ancestral components of the 40 individuals and visualized using R. Considering the complex history of the Tarim River, such as multiple water diversion projects, there may be more than four populations. Therefore, the default K value was set as 1–9, the bootstrap value was set as 1000 and other parameters were set as default. The optimal K value was determined by a cross-validation error of the software. 

### 2.4. SSR Loci Detection and SSR Marker Development

Microsatellite mining (http://pgrc.ipk-gatersleben.de/misa/ (accessed on 9 November 2020)) was performed using the Microsatellite (MISA) identification tool, Primer3 V2.3.6 [12] for each SSR primer pair at default Settings design, the size of the PCR products between 100~300 bp. Locus polymorphism screening mainly included singleness and polymorphism screening. Singleness was determined by agarose gel after PCR and whether the product is single or not. The polymorphic screening was performed by agarose electrophoresis after PCR with fluorescent primers to determine whether the peak types were qualified or polymorphic.

## 3. Results

### 3.1. RAD Sequencing and Data Quality

Forty *H. leucisculus* were sequenced using RAD-seq. The sequence results showed that 632,037,408 clean reads were obtained, with an average number of 15,800,935 samples, and the average quality of Q20 was 96.91%. The average quality of Q30 was 91.57%. The average GC content was 39%, indicating that the difference before and after filtration is very small. Q20 and Q30 were both at a high level, and GC content was low, which proved that the sequencing quality was up to standard, the base error rate was very low and the sequencing information was reliable (Table 1).

**Figure 2:**
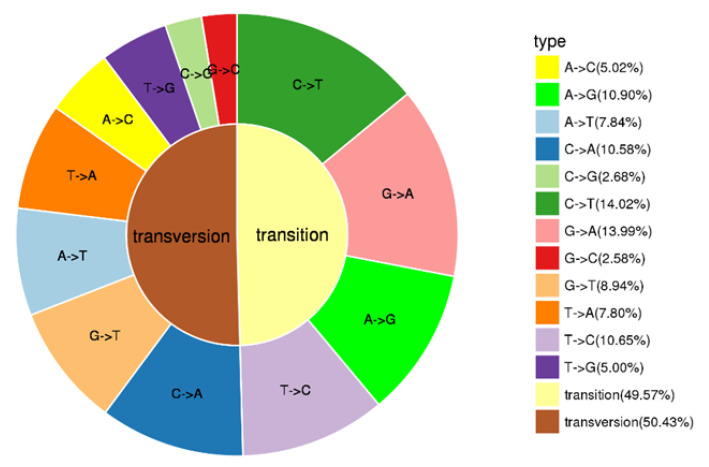
Types of SNPs.

Population SNP spectrum analysis (Figure 2) showed that SNP mutations could be divided into 12 categories, with A/C (10.90%), C/A (10.58%), C/T (14.02%), G/A (13.99%) and T/C (10.65%) as the main types of SNP mutations. Transversions (Tv) was larger than transition (Ti), accounting for 50.43% of SNP, and the ratio of Ti to Tv is 0.98.

### 3.2. Genetic Diversity and Genetic Differentiation Analysis

The genetic diversity of 40 *H. leucisculus* populations was analyzed. The results showed that the population genetic diversity level of *H. leucisculus* was low. The nucleotide diversity (Pi) of the populations ranged from 0.000964 to 0.001144, which was highest in TTMH. The observed heterozygosity (Ho) across all populations ranged from 0.0593 to 0.0774, with an average of 0.0672. Ho was lowest in YBZ (0.0593) and highest in TTMH (0.0774). Expected heterozygosity (He) ranged from 0.1925 to 0.2055. *H_e_* was lowest in YBZ (0.1925) and highest in TTMH (0.2055). The individual inbreeding coefficients (*F*_IS_) of the populations ranged from 0.402 to 0.7016 (Table 2). The Ho of the four populations was lower than the He, and *F*_IS_ was greater than 0, indicating that heterozygote deletion existed in *H. leucisculus* population. Pi was lower than 0.005, which showed that the genetic diversity of *H. leucisculus* population was relatively weak. TTMH had the highest Ho, He and Pi, indicating that the population had the highest genetic diversity. 

The population pairwise *F*_ST_ values varied from 0.231 to 0.258 (Table 3). Significant genetic differentiation was observed in all population pairs. The lowest *F*_ST_ values were between QL and TTMH and highest *F*_ST_ values were between THY and YBZ. In addition, to detect genetic variance components within and among populations, AMOVA was conducted (Table 4). AMOVA showed that the genetic variation within populations accounted for 92.31% of total variation, and 7.69% of variation was distributed among populations.

### 3.3. Population Structure Analysis

**Figure 3:**
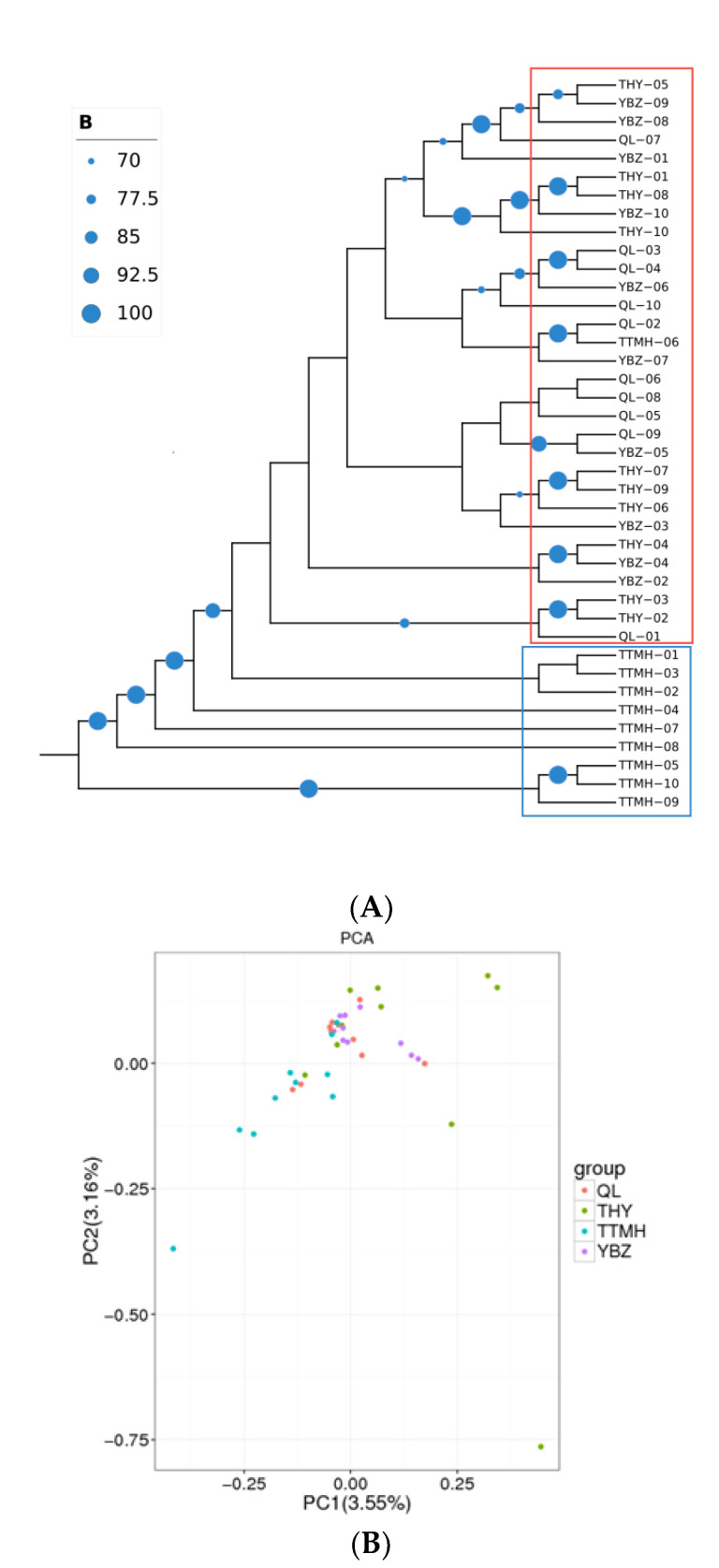
The NJ tree of 40 individuals based on SNPs (**A**) and the PCA plotting of the individuals from different locations (**B**). The size of the blue circle at the node represents the size of the bootstrap value and squares with different colors represent different clusters (blue: cluster 1; red: cluster 2) in (**A**).

According to the SNP information generated form RAD-seq, the NJ tree was performed in the 40 individuals (Figure 3A). The NJ tree was supported by bootstrapping (>70%; Figure 3A). The results showed that the forty individuals could be grouped into two clusters (upper–middle and downstream populations). TTMH formed a cluster (cluster 1), and the other populations formed a large group (cluster 2). However, to some extent some individuals from TTMH can be clustered into the large cluster 2 (QL, THY, YBZ) together, which indicated that TTMH did not split as a whole and the four populations had a contact. The results of the PCA scatter plot, of which principal factor 1 explained 3.55% and principal factor 2 explained 3.16% of the overall variance, were consistent with NJ tree (Figure 3B). The PCA indicated that QL, THY and YBZ were clustered closely and showed a highly mixed state. In addition, the individuals from TTMH clustered together with other populations, but they had a bigger geographic distance than the other populations. The results of PCA were consistent with the NJ tree.

**Figure 4:**
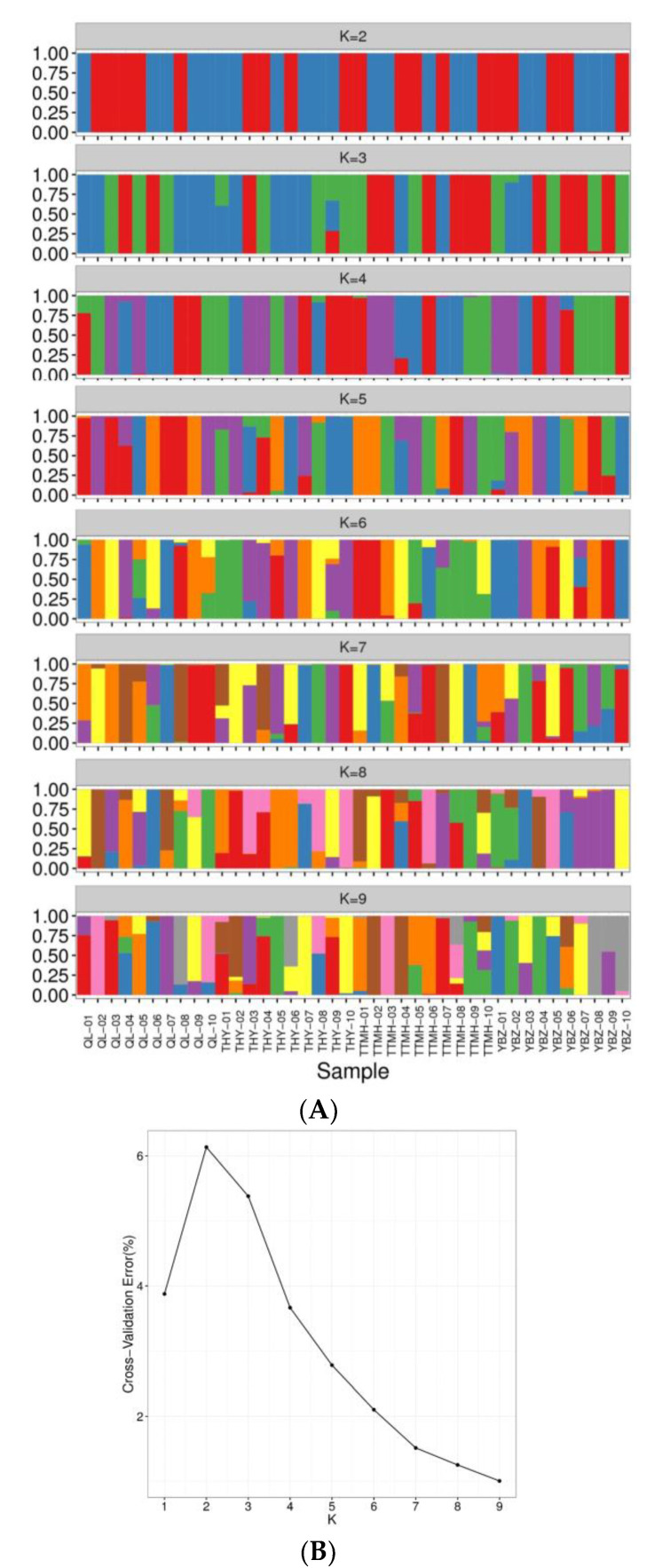
Analysis of the population structure from four locations. (**A**): Population structure of 40 individuals from different sites. (**B**): The admixture validation error rate corresponding to the different K values.

To further reveal the genetic structure of 40 individuals, the cluster analysis was conducted (K = 1~9). The CV error showed the lowest value with K = 9 and indicated that the optimal cluster number was nine genetic clusters (Figure 4B). When the K value was 3, the component of ancestry of most individuals from TTMH and YBZ was similar compared with QL and THY. When the K value was 6, the component of ancestry information of QL, THY and TTMH was similar except YBZ. Notably, TTMH formed a cluster compared with the other locations, which corresponded with the results of NJ tree and PCA plotting. When the K value was 9, several individuals had the admixture ancestry composition, such as THY-02, TTMH-08 and TTMH-10, which indicated that there could have been four different ancestries (Figure 4A).

### 3.4. Frequency of SSRs in H. leucisculus

**Figure 5:**
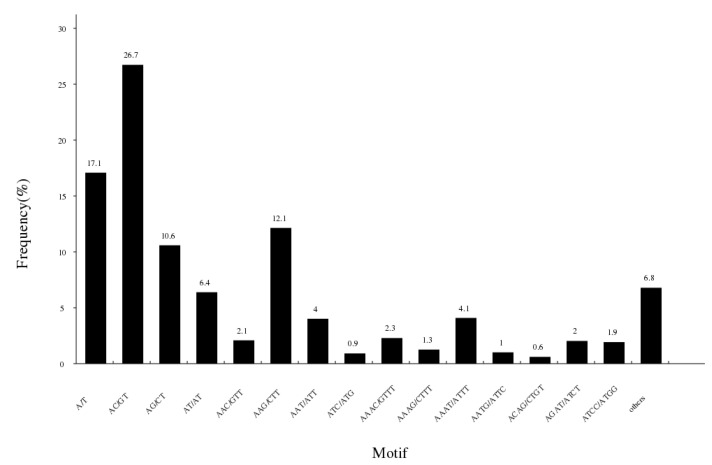
Characterization and frequency of microsatellites with different motif sizes.

The study detected a total of 147,705 potential simple sequence repeats in 1,533,492 fragments (Table 5). The unit size of SSRs varied from 1 to 6, including 25,576 (17.32%) mono-nucleotide motifs, 64,803 (41.68%) di-nucleotide motifs, 29,974 (20.29%) tri-nucleotide motifs and 22,566 (15.28%) tetra-nucleotide motifs. There were 3947 (2.67%) penta-nucleotide motifs and 839 (0.57%) hexa-nucleotide sequences. The motif (AC/GT) was the most abundant repeating type, accounting for 26.7%, was followed by A/T (25242, 17.1%), AAG/CTT (17942, 12.1%), AG/CT (15646, 10.6%), AT/AT (9439, 6.4%) and AAAT/ATTT (6046, 4.1%) (Figure 5).

### 3.5. SSR Marker Development and Characterization

The Primer3 V2.3.6 was used to develop SSR primer pairs using 147,705 sequences containing SSRs (Table 5). Finally, 22,651 pairs of primer pairs were successfully designed, and 17,211 locus-specific primer sets were retained after strict filtering.

In order to verify the value of these primers via PCR, 10 primers pairs were randomly selected and synthesized to substantiate in forty *H. leucisculus* individuals. Among the 10 primers, 6 pairs of *H. leucisculus* genomic DNA were successfully amplified by PCR, while the other 4 pairs of primers could not obtain PCR products (Table 6). Most successful primer pairs amplified expected size bands.

This proved that the ability of high-throughput sequencing identifies genes in non-model organisms [13]. These results will provide valuable tools for future genetic studies at *H. leucisculus*.

## 4. Discussion

RAD-seq technology includes the lower cost acquisition of DNA sequence data from multiple sites in the genome, simpler library preparation processes, the simplified management of genomic data and improved pipelines for data processing and analysis [14]. It can be used to identify and genotype large numbers of SNPs [15]. SNP refers to the DNA sequence polymorphism formed due to nucleotide variation. SNP has the characteristics of a large number, wide distribution and high diversity in biological individuals and plays an important role in the fields of life science and molecular breeding [16]. Though RAD-seq requires considerable sequencing effort per individual compared with ddRAD, the values of commonly used summary statistic, Pi, Tajima’s D and *F*_ST_ of RAD-seq has lower deviation than ddRAD [17,18]. In this study, forty *H. leucisculus* individuals were sequenced using RAD-seq and a total of 7,291,260 high-quality SNPs were detected, which provided the basis for molecular marker development, population differentiation and the invasion analysis of the Tarim River *H. leucisculus* in Xinjiang.

Genetic diversity research can reveal the status quo of species germplasm resources, genetic diversity and phylogenetic evolution of species [19]. Due to the lack of the genome-wide sequence of *H. leucisculus*, the RAD-seq method was chosen to analyze the population genetic diversity of *H. leucisculus* in this study. According to Ichikawa et al. the genetic diversity of *H. leucisculus* populations in Tarim River was low (Pi < 0.005) [20]. Genetic diversity is one of the most important factors affecting the success of invasive species [21]. It is traditionally believed that rich genetic diversity will help alien species adapt to new habitats and establish and maintain new population sizes [22]. We examined the relevant information that the Pi of *H. leucisculus* in the Huaihe River basin was low, while that in the Yangtze River was high (Pi = 0.03289) [23,24]. The reason could be that compared with the Yangtze River basin, the unstable water environment in the Tarim and Huaihe River basins makes fish resources vulnerable to environmental changes, leading to a decrease in genetic diversity [21]. In addition, *H. leucisculus* may have been affected by the founder effect during the invasion of the Tarim River, resulting in lower genetic diversity than in the Yangtze River basin [25,26]. The *F*_IS_ in populations can directly indicate the loss or surplus of heterozygotes. The *F*_IS_ values for the four populations were greater than 0, indicating that the *H. leucisculus* population had different degrees of heterozygote loss. Combined with *H_o_* < *H_e_*, there was a certain degree of inbreeding. The *F*_ST_ value has three levels of different genetic differentiation: moderate differentiation was 0.05 to 0.15, larger differentiation was 0.15 to 0.25 and high differentiation was greater than 0.25 [27]. In fact, RAD-seq has the drawbacks that missing data tends to inflate estimates of *F*_ST_ [17]. Therefore, the genetic differentiation of *H. leucisculus* may be at a moderate level. Combined with low genetic diversity, the high *F*_ST_ values (*F*_ST_ = 0.243) among these populations may be caused by habitat fragmentation in the Tarim River basin [28,29,30]. In addition, we noticed that the genetic differentiation of *H. leucisculus* was lower in the lower reaches than in other regions. This could be that the upstream terrain is more rugged than the downstream. Rugged reaches promote genetic differentiation, and gentle reaches are more conducive to gene flow [31].

Combined with the results of NJ tree and PCA, the individuals from TTMH showed differences and had a bigger geographic distance than the others. The genetic differentiation pattern of fish is usually consistent with the water system pattern of distribution [32,33]. It could be explained by the reason that due to the large-scale diversion and interception of water in the middle and upper reaches of the Tarim River, the lower reaches of Tarim River have been cut off [28,34,35]. It was not until 2000~2006 that the Lower Tarim River Emergency Water Transfer transferred water from Bosten Lake to Taitema Lake that this phenomenon was alleviated [36,37]. Therefore, geographic isolation led to a clear differentiation of TTMH populations. In addition, the result of population structure analysis showed that the degree of mixed blood among individuals was high, indicating frequent gene exchange. This could be because Taitema Lake is located at the end of the Tarim River, and the alluvial plain formed by three water systems of Tarim River, Qarqan River and Altun Rivers, with frequent water exchange [35]. There was increased gene flow between populations during floods [38,39,40].

*H. leucisculus* is an invasive species in the Tarim River system of Xinjiang. According to the population structure analysis, it was inferred that the origin of the four sites was the same species. Compared with the others, TTMH had some genetic differences. Dong et al. showed that human impact index (HII) was the most important factor affecting *H. leucisculus* invasion among the determinants of *H. leucisculus* invasion [41]. The water diversion project transferred water from Bosten Lake to Taitema Lake. In 1958, Bosten Lake introduced *Cyprinus carpio*, *Carassius auratus*, *Hypophthalmichthys molitrix* and *Hypophthalmichthys nobilis* directly or indirectly from the Yangtze River basin for economic benefits and *H. leucisculus* and Bosten male fish in the pond dominated [42,43]. High natural diffusion and human translocation are conducive to gene flow and high genetic diversity among river basins, which might help retain the invasive potential [38]. Therefore, it is speculated that *H. leucisculus* from Bosten Lake entered Taitema Lake to breed and then expanded into the Tarim River.

SSR markers are one of the most widely used molecular markers and play an important role in genetic research. Traditional SSR development methods often needed to construct the genome enrichment library, and the hybridization screening and sequencing processes were time-consuming and laborious. Today, the development of high-throughput sequencing technology and the sharp reduction of cost create better conditions for the development of SSR markers, which have the advantages of short development cycle, high yield and excellent flexibility [44]. In recent years, the technology of developing SSR markers using RAD to simplify genome sequencing has become increasingly mature, and there are more and more reports concerning it. Qu et al. used RAD-Seq to perform simplified genome sequencing on Indonesian tigerfish (*Datnioides Pulcher*), and a total of 26,359 SSR loci were detected through sequence analysis [45]. Wang et al. identified a total of 466 983 SSR loci by analyzing the RAD simplified genome sequencing data of *Pelteobagrus Wallis* [46]. At present, there are few reports about the development of *H. leucisculus* SSR markers. In this study, 22,651 SSR primers were designed to meet the primer development conditions by analyzing the simplified genome sequence of *H. leucisculus*. These results provide a basis for the development of *H. leucisculus* new primers.

## 5. Conclusions

In the study, low levels of genetic diversity were observed among four populations in the Tarim River. Major genetic variations originated from within populations variation and moderate genetic differentiation among these populations was observed. The four populations were able to be separated into two clusters (upper–middle and downstream populations), and the individuals from TTMH clustered and had a bigger geographic distance than the others. There is the probability that the *H. leucisculus* from Bosten Lake entered Taitema Lake to breed and then expanded into the Tarim River due to the water diversion projects in location. In addition, 147,705 SSRs loci were detected and 22,651 SSR primer pairs were developed. This study provides valuable genetic information for the management of wild populations, marker-assisted selection and resource exploitation of *H. leucisculus*.

## Figures and Tables

**Table 1:** Statistics of genomic sequences generated by RAD-seq.

Sample	Before Filter					After Filter				
	Raw Read Data	Clean Date (bp)	Q20 (%)	Q30 (%)	GC (%)	High-Throughput Reading	HQ Clean Data (bp)	Q20 (%)	Q30 (%)	GC (%)
THY	7,680,728	2,242,772,518	96.49	91.01	39.66	7,352,661	2,146,939,680	96.81	91.41	38.86
YBZ	7,215,133	2,106,818,865	97.03	91.88	38.83	7,084,553	2,068,663,228	97.17	92.05	38.74
QL	8,504,442	2,483,297,035	96.27	90.56	39.95	8,247,451	2,408,227,372	96.53	90.86	39.36
TTMH	8,201,568	2,394,857,739	96.96	91.78	39.41	8,031,269	2,345,097,675	97.11	91.96	39.29

**Table 2:** The statistical values of genetic diversity among different clusters of *H. leucisculus*.

Population Name	Number of Individuals	*H_o_*	*H_e_*	Pi	*F*_IS_
THY	10	0.0677	0.2010	0.001012	0.60082
YBZ	10	0.0593	0.1925	0.000964	0.63508
QL	10	0.0645	0.1983	0.001097	0.60286
TTMH	10	0.0774	0.2055	0.001144	0.54973

**Table 3:** Pairwise *F*_ST_ among four populations of *H. leucisculus*.

Population Name	THY	YBZ	QL	TTMH
THY	0	0.258	0.249	0.235
YBZ	0.258	0	0.251	0.237
QL	0.249	0.251	0	0.231
TTMH	0.235	0.237	0.231	0

**Table 4:** Analyses of molecular variance (AMOVA) of *H. leucisculus*.

Source of Variation	d.f.	Variance Components	Percentage of Variation
Among populations	3	0.000402	7.69
Within populations	36	0.028631	92.31
Total	39		100

**Table 5:** Detection results of SSR loci in *H. leucisculus*.

Item	Number
Total number of sequences examined	13,537,492
Total size of examined sequences	604,493,653
Total number of identified SSRs	147,705
Number of SSR containing sequences	126,849
Number of sequences containing more than 1 SSR	17,352
Number of SSRs present in compound formation	11,698

**Table 6:** The messages of 6 working SSR primer pairs from 10 synthesized primers.

ID	Forward Primer (5′-3′)	Reverse Primer (5′-3′)	Tm (°C)	Repeat Motif	Product Size
P1	CCTCACTGAACCCTTAACGC	GCTTGGAAGAACATTGGAGC	60	(TG)_6_	223
P2	TCTGGTTGATTGGGAAAAGTG	CACTAACAGGTGCCGTGATG	60	(AC)_46_	228
P3	GGTCACCCCAGACATTGTTC	GCAGGAGAAAGCACACTTCC	60	(AAG)_11_	196
P4	ACATTGTGGGGACATTTGGT	GGTAGGGGTAGTGTAGGGGG	60	(CAT)_11_	144
P5	TGCTGACATGTTTGCATTTG	ATTTTGCTTTGTGAAACCGC	59	(CACGTT)_18_	265
P6	TTCATGATTTTCCCTGTCTGC	AGCTGCAGGATCTGATGGAT	60	(ATAAC)_22_	231

## Data Availability

The raw genome sequencing datasets generated during the current study have been submitted to the NCBI Sequence Read Archive (SRA) and are available online at http://www.ncbi.nlm.nih.gov/bioproject/848648 (accessed on 30 June 2022).
